# Focal Segmental Glomerulosclerosis: Comprehensive Review and Exploration of the Dual Potential of Cyclodextrins in Therapeutic Optimization

**DOI:** 10.3390/ijms26188760

**Published:** 2025-09-09

**Authors:** Filipa Mascarenhas-Melo, Bruna Martins, Inês Monteiro, Alka Lohani, Karolline Krambeck

**Affiliations:** 1 Higher School of Health, Polytechnic Institute of Guarda, Rua da Cadeia, 6300-307 Guarda, Portugal; brunapmartins2002@gmail.com (B.M.); inesginjeira@gmail.com (I.M.); 2 BRIDGES—Biotechnology Research, Innovation and Design for Health Products, Polytechnic University of Guarda, Avenida Dr. Francisco Sá Carneiro, No. 50, 6300-559 Guarda, Portugal; 3 Amity Institute of Pharmacy, Amity University Uttar Pradesh, Noida 201313, India; alkalohani06@gmail.com

**Keywords:** focal segmental glomerulosclerosis, pathophysiology, conventional treatments, drug delivery, cyclodextrins

## Abstract

Focal segmental glomerulosclerosis (FSGS) is a histopathological pattern of segmental glomerulosclerosis that arises from diverse primary and secondary causes. Primary (idiopathic) FSGS is rare and is often linked to intrinsic podocyte injury, while secondary forms are more prevalent and may reflect adaptative, toxic, genetic, or viral etiologies. This pattern of injury can lead to progressive renal dysfunction and, in some cases, end-stage kidney disease. The pathophysiology is multifactorial and includes direct podocyte injury (e.g., genetic defects, mechanical or toxic injury), immune-mediated processes (e.g., circulating permeability factors, inflammatory mediators), and metabolic disturbances. In particular, disturbance of lipid metabolism, including intracellular cholesterol accumulation in podocytes, have been implicated as a contributory mechanism in podocyte dysfunction and progression of disease in proteinuric/nephrotic presentations and in specific disease subtypes. Diagnosis relies on clinical assessment, laboratory testing, and histological examination, with kidney biopsy remaining the gold standard. Conventional treatments include corticosteroids, and other immunosuppressants when indicated, and measures to reduce proteinuria and control blood pressure, but the therapeutic response is variable and many patients show progression, highlighting the need for more effective and novel therapeutic approaches. Cyclodextrins (CDs), widely used as drug carriers to enhance solubility, can also mobilize and promote efflux of cholesterol from cells. Preclinical studies show that CDs reduce renal lipid accumulation and ameliorate podocyte injury in experimental models, supporting the idea that CDs could have a dual role as drug carriers and as direct modulators of lipid-related podocyte injury in lipid-associated forms of FSGS. Given the limited direct clinical data in FSGS, in this article we discuss the biological rationale, preclinical evidence, and remaining knowledge gaps for exploring CDs as an innovative therapeutic strategy.

## 1. Introduction

Chronic kidney disease (CKD) is a public health problem, affecting around 10–15% of the pediatric population and 3% of the adult population in the United States of America (USA) [1]. One of the main causes of CKD is focal segmental glomerulosclerosis (FSGS) [2,3,4,5]. It is also known that this pathology accounts for between 10 and 20% of glomerulopathies diagnosed by kidney biopsy in adults in Europe [6]. In 2022, the United Kingdom recorded around 26,000 new diagnosed cases of FSGS [7].

According to the Food and Drug Administration (FDA), FSGS is considered a rare kidney disease [8]. It is characterized by the loss of renal podocytes and the progressive sclerosis of some glomeruli (focal), affecting only specific parts (segmental) of them [5,9,10,11]. FSGS can be classified as primary or secondary, the latter being subdivided into adaptive, genetic, virus-mediated forms, associated with the use of drugs or psychoactive substances and the presence of risk variants in the Apolipoprotein L1 (APOL1) gene [4,8,12,13,14].

Podocytes are terminally differentiated epithelial cells that form the visceral layer of Bowman’s capsule and are a critical component of the glomerular filtration barrier [15]. Their interdigitating foot processes and slit-diaphragm complexes maintain size- and charge-selective filtration; disruption of podocyte structure or function leads to foot-process effacement and proteinuria [16]. Structural or functional podocyte injury, whether from genetic defects, mechanical/adaptive stress, toxins, immune-mediated circulating factors, or metabolic perturbations, contributes to podocyte depletion and the development of segmental glomerulosclerosis in FSGS [17,18]. Lipid/cholesterol dysregulation within podocytes has been implicated as one of several metabolic mechanisms that can aggravate podocyte dysfunction in proteinuric states [19].

The incidence and prevalence of FSGS are difficult to estimate, since diagnosis often depends on performing a kidney biopsy, which can delay detection of the disease. Nevertheless, it is recognized that the prevalence of FSGS has been increasing globally [8].

A population-based case series in Southern California (2000–2011) identified 2501 adults with biopsy-proven primary glomerulonephropathies; focal segmental glomerulosclerosis (FSGS) accounted for 38.9% of cases. Over the study period, overall rates of primary glomerulonephropathies increased, driven mainly by FSGS, which was estimated at 2.7 cases per 100,000 person-years. FSGS was more frequent in men (1.5 times higher) and showed a higher proportion among black patients (6.8 cases per million) compared with white patients (1.9 cases per million) [20,21].

In Europe, it is estimated that FSGS accounts for around 9% of kidney biopsies carried out on adults [22]. The causes of the increase in the incidence and prevalence of FSGS remain unclear, but factors such as greater access to diagnosis, as well as the increase in obesity and chronic low-inflammation, particularly in metabolic syndrome, type 2 diabetes, and obesity-related FSGS, are likely contributors [23,24,25].

The prognosis of FSGS is variable and depends on multiple factors, including the levels of proteinuria and plasma creatinine, the morphological subtype of the disease, the degree of renal fibrosis, the response to treatment and the underlying causes. Early diagnosis, combined with timely treatment, is decisive in improving clinical outcomes and slowing down the progression of the disease [8].

Conventional therapy for FSGS is based on antihypertensive and immunosuppressive drugs, including monoclonal antibodies, aimed at reducing proteinuria and inflammation. However, these approaches have proved to be insufficient in many cases, which has motivated the scientific community to study the disease further and explore new therapeutic strategies [26].

Nanotechnology has played an important role in this area, particularly through the development of innovative therapeutic systems such as cyclodextrins (CDs). CDs are hydrophilic cyclic oligosaccharides obtained from the enzymatic degradation of starch, with a truncated cone-shaped structure. They have been widely studied as drug delivery systems due to their ability to increase the solubility, permeability and stability of bioactive compounds without compromising the integrity of the cell membrane [27]. In addition to their role as transporters, CDs have also demonstrated intrinsic properties, namely, the ability to sequester cholesterol accumulated in the tissues, an aggravating factor in the kidney damage observed in FSGS. This dual action makes CDs particularly promising in the development of new treatment strategies for this pathology. The main CDs used are α, β and γ, whose therapeutic application will be discussed throughout this review [28,29].

In this context, the aim of this paper is to clarify the pathophysiology of FSGS, the conventional treatments used and to explore new therapeutic approaches using CDs, highlighting both their role as advanced drug delivery systems and their intrinsic therapeutic action, namely, their ability to sequester excess cholesterol in renal tissues. To this end, concrete examples of studies using CDs, alone or in combination with drugs, will be presented, allowing promising approaches to be identified for the treatment of FSGS. The results gathered here may help to expand knowledge in this area, guide future research and support more effective therapeutic decisions.

## 2. Pathophysiology of FSGS

FSGS is a relatively rare renal pathology whose clinical complexity and heterogeneity contribute to difficulties in its diagnosis, treatment and pathophysiological understanding. Although less studied compared to other more prevalent nephropathies, scientific interest in FSGS has been growing, mainly due to its significant impact on kidney function and patients’ quality of life.

The exact etiology of FSGS remains unclear [30], but it is believed to be multifactorial, involving genetic, environmental and immunological factors. The main risk factors identified include male gender, black ethnicity, a family history of the disease and the consumption of nephrotoxic substances such as heroin [31].

**Figure 1:**
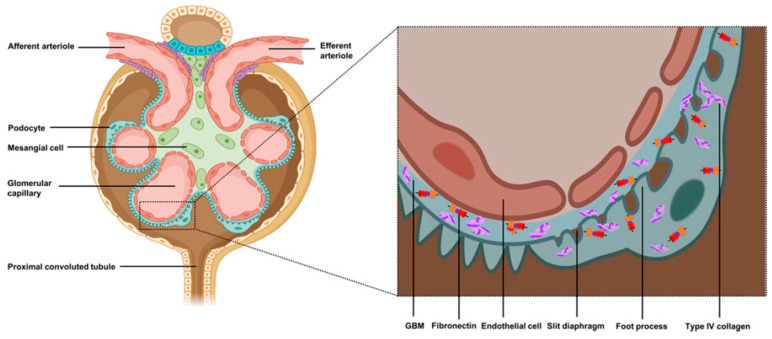
Structural organization and function of podocytes. Together with the endothelial cells of the capillaries and the glomerular basement membrane (GBM), podocytes constitute the fundamental barrier to glomerular filtration. Their cytoplasmic projections, known as pedicels, are interconnected by a specialized structure called the filtration diaphragm, which is essential for ensuring the selectivity of the glomerular barrier. In addition to their structural role, podocytes actively participate in maintaining the GBM, contributing to its composition by synthesizing type IV collagen and fibronectin, and ensuring the metabolic balance of this component. Adapted from [33].

From a structural point of view, podocytes, highly differentiated epithelial cells, play a crucial role in maintaining the integrity of the glomerular filtration barrier [32]. They act together with capillary endothelial cells and the glomerular basement membrane, making up the three main components of the protein filtration barrier [33] (Figure 1). Podocyte damage or dysfunction compromises this selective barrier, allowing for the abnormal passage of plasma proteins, such as albumin, into the urine, a phenomenon known as proteinuria, often identified by the presence of frothy urine [9]. The loss of podocytes is associated with their limited regenerative capacity. When cell depletion occurs, the remaining podocytes compensate through hypertrophy, trying to cover a larger area of the glomerular capillary surface [33]. However, this adaptive mechanism ends up being insufficient in the long term, contributing to the progression of segmental sclerosis of the glomeruli.

**Figure 2:**
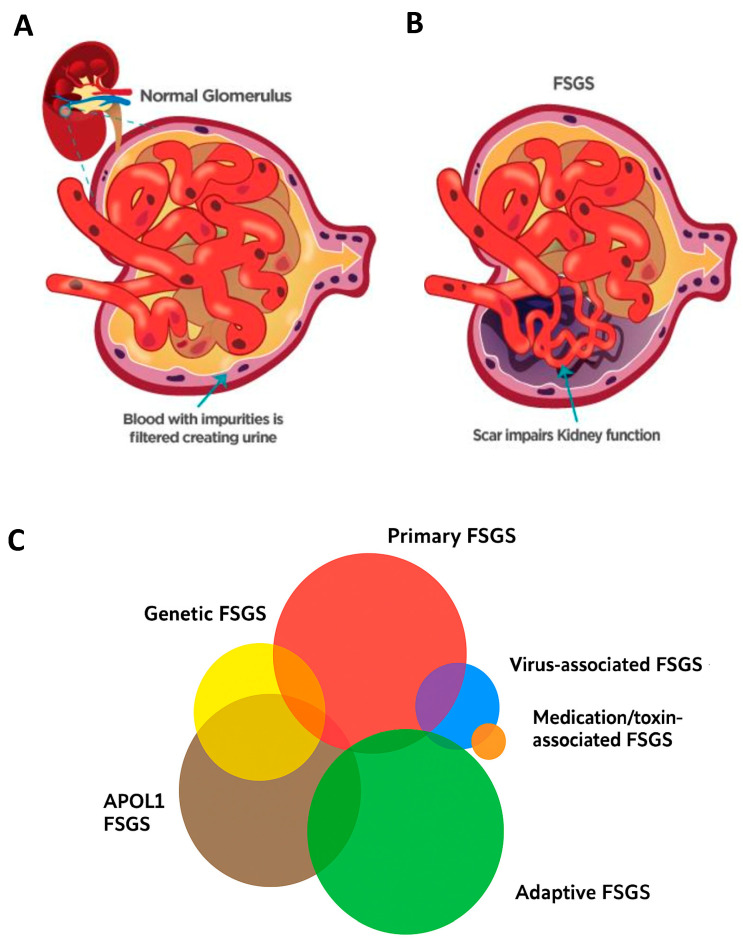
Schematic representation of (**A**) Normal Glomerulus, (**B**) Glomerulus with FSGS. (**C**) Different types of FSGS: the size of each cloud represents the approximate relative distribution of these variants in the US population. Adapted from [35].

FSGS can be categorized into two main forms: primary, of idiopathic etiology, and secondary, which results from identifiable causes. The secondary forms are subdivided into several categories according to the underlying mechanism: adaptive (associated with hemodynamic overload or glomerular hypertrophy), genetic (resulting from mutations in genes that code for podocyte structural proteins), virus-mediated (such as Human Immunodeficiency Virus (HIV) or Parvovirus B19), induced by drugs or recreational substances and associated with risk variants of the APOL1 gene (Figure 2) [8,12,14,34].

### 2.1. Primary FSGS

Primary FSGS, classified as idiopathic after exclusion of secondary causes, remains an important cause of nephrotic syndrome, especially in children and adults. Secondary FSGS, resulting from identifiable causes such as adaptive responses to reduced nephron mass, viral infections, drugs, or genetic mutations, is generally more common than primary FSGS [36].

In individuals of African ancestry, genetic forms of FSGS linked to APOL1 risk variants are the predominant subtype, accounting for a substantial proportion of cases previously labeled as primary FSGS [37,38].

Therefore, the epidemiology of FSGS varies significantly with underlying etiology and patient population [39,40].

### 2.2. Secondary FSGS

#### 2.2.1. Adaptive FSGS

Adaptive FSGS occurs as a response of the kidney to functional overload, this overload being caused by conditions that lead to a reduction in the number of functional nephrons or an increase in the overload on the remaining nephrons [8]. Some conditions associated with this overload include systemic diseases such as Diabetes Mellitus, hypertension and obesity [32], and other diseases such as glomerular hypertension, hyperfiltration [11], renal aplasia, hypoplasia or dysplasia, renal artery stenosis, vascular disease [32], sickle cell anemia and atherosclerosis [41].

#### 2.2.2. Genetic FSGS

As a podocytopathy characterized by lesions in the podocytes, FSGS can have a genetic origin through mutations in various genes that encode proteins essential for the structure and function of these specialized cells of the glomerulus. These mutations can compromise various proteins that are fundamental to the integrity and functionality of podocytes. Alteration of the genetic sequence leads to the production of dysfunctional proteins or their absence, which results in structural instability, loss of selectivity of glomerular filtration and progression of kidney disease. Thus, the direct relationship between genetic mutations and protein function in podocytes is a determining factor in the development of FSGS. Table 1 shows some of the genes and respective proteins involved in genetic FSGS [10].

#### 2.2.3. Virus-Mediated FSGS

FSGS can arise as a result of viral infections that trigger damage to the renal glomeruli, resulting in progressive scarring of the renal tissue. Various viruses have been associated with the development of this pathology, such as HIV, which attacks tubular and glomerular epithelial cells, and Parvovirus B19, which acts directly on podocytes. Hepatitis B and C viruses also target podocytes, contributing to glomerular dysfunction. Cytomegalovirus affects multiple renal cell types, including tubular, epithelial and endothelial cells, causing necrosis, inflammation and cell proliferation. The Epstein–Barr virus, on the other hand, can affect podocytes or other kidney cells, also causing structural damage that favors the progression of FSGS [8,12,42].

#### 2.2.4. FSGS Associated with the Use of Drugs or Psychoactive Substances

Some drugs and psychoactive substances can induce FSGS through direct mechanisms, such as podocyte toxicity, or indirect mechanisms, through hemodynamic or metabolic alterations.

In direct mechanisms, the agents act directly on the podocytes or other components of the kidney tissue, causing cell damage and subsequent glomerular sclerosis. Examples include non-steroidal anti-inflammatory drugs, anabolic steroids, interferons (IFN-α, -β and -γ), immunosuppressants such as tacrolimus and sirolimus, bisphosphonates such as pamidronate, lithium, anthracyclines such as doxorubicin and some drugs such as heroin and cocaine [12,42].

On the other hand, indirect mechanisms do not cause immediate glomerular damage, but jeopardize kidney function through systemic alterations. Hemodynamic mechanisms include hypertension, which by increasing the pressure in the renal vessels can cause endothelial damage and progressive glomerular sclerosis; chronic use of diuretics or antihypertensives, which can affect renal perfusion; excessive consumption of salt or sugar, which alters the osmotic balance and pressure, impairing renal blood flow; endothelial dysfunction, often observed in hypertensive states and Diabetes *Mellitus*, which compromises the integrity of the vascular barrier, favoring glomerular inflammation.

Metabolic mechanisms refer to alterations in systemic metabolism that directly affect kidney function. Examples include Diabetes *Mellitus*, in which persistent hyperglycemia promotes structural changes in the glomeruli; dyslipidemia, which can lead to lipid deposition in the renal arterioles, affecting glomerular perfusion; and hyperuricemia, in which uric acid crystals can induce inflammation and glomerular damage.

The proper functioning of the glomeruli depends not only on efficient renal perfusion, but also on the integrity of the cells that make up the filtration barrier. Thus, any factor that jeopardizes these elements can trigger a process of glomerular scarring, culminating in the progressive loss of renal function and, eventually, chronic renal failure [8,10].

It should be noted that, in most cases, drug-induced FSGS is potentially reversible. Suspension of the causative agent, combined with treatment with glucocorticoids, can allow renal function to recover [42].

#### 2.2.5. Apolipoprotein L1 (APOL1)-Associated FSGS

APOL1 is a plasma protein involved in lipid transport and the innate immune response, playing an important role in the body’s defense against parasites, particularly *Trypanosoma brucei*, the etiological agent of sleeping sickness. However, specific genetic variants of the APOL1 gene, identified as G1 and G2, are associated with a significantly increased risk of developing kidney diseases, including FSGS, particularly in individuals of African descent [18].

The G1 variant corresponds to a compound mutation resulting in the substitution of two amino acids in the APOL1 sequence (“nonsense” mutation). The G2 variant, on the other hand, results from a deletion of six base pairs, causing the loss of two amino acids in the C-terminal helix region of the protein. Both alterations compromise the normal function of APOL1.

Although these variants provide protection against infection by *Trypanosoma brucei*, they also confer greater susceptibility to the development of kidney pathologies. APOL1 dysfunction negatively affects lipid homeostasis and compromises podocyte function, leading to their necrosis and, consequently, the progression of FSGS [43].

In the USA, it is estimated that around 40% of cases of end-stage renal disease attributed to FSGS occur in black individuals, and that approximately 72% of these cases are associated with the presence of risk variants in the APOL1 gene [8].

It is important to clarify that APOL1-associated FSGS is considered a genetic form of the disease due to the direct involvement of inherited risk alleles. However, it is often discussed separately from other genetic causes because of its unique epidemiological distribution, predominantly affecting individuals of African ancestry, and its distinctive pathogenic mechanisms linked to APOL1 variant expression. This separation allows for a clearer understanding of its population-specific impact and guides targeted therapeutic approaches [39].

## 3. Clinical Manifestations and Diagnosis of FSGS

FSGS can be asymptomatic or symptomatic, with proteinuria being the most common initial clinical manifestation, especially in cases of primary FSGS. When left untreated, proteinuria can progress to CKD and, in more serious situations, kidney failure, which may require renal replacement therapy such as dialysis or transplantation [31,33].

Other clinical signs and symptoms frequently observed include frothy urine, hypertension, progressive reduction in kidney function, especially in advanced stages of the disease, generalized oedema, weight gain, fatigue, uremia, hypoalbuminemia, hyperlipidemia and, occasionally, microscopic hematuria [3,33,44].

Early diagnosis of FSGS is essential to guide the choice of therapy and estimate the prognosis. However, in the early stages, many patients have no obvious symptoms, which can delay diagnosis [44].

The diagnostic process includes a combination of laboratory tests, urinary analyses, imaging techniques, renal biopsy and, in some cases, genetic testing (Table 2). However, only renal biopsy can definitively confirm the final diagnosis [45], by observing the characteristic sclerotic lesion of FSGS. Nevertheless, complementary tests play an important role in clinical suspicion and in excluding other nephropathies [8].

The combined use of three types of microscopy, optical, scanning electron and transmission electron, is crucial for differentiating FSGS from Minimal Lesion Disease (MLD) as both are characterized by significant proteinuria [8].

However, only optical microscopy can identify the morphological variants of FSGS, which differ in terms of the pattern of glomerular damage and the pathophysiological mechanism. The Columbia Classification, proposed in 2004, categorizes FSGS into five distinct variants: unspecified, collapsing, perihilar, cellular and tip lesion (Table 3) [10,13,46].

## 4. Conventional Treatment of FSGS

There is currently no proven effective treatment for curing FSGS, as the glomerular scarring process is considered irreversible [47].

Therefore, the therapeutic strategies currently adopted are mainly aimed at symptomatic control, slowing down the progression of the disease and, whenever possible, addressing its underlying causes, with the aim of preserving kidney function and improving patients’ quality of life [8].

Selecting the most appropriate therapy first requires identifying the etiology of FSGS, distinguishing between primary and secondary forms. Secondary forms can include adaptive, genetic, viral etiologies, those associated with the use of drugs or psychoactive substances, as well as alterations associated with APOL1. In addition, it is essential to consider individual factors such as the patient’s age and the presence of relevant comorbidities [8].

The treatment of FSGS is currently based on three main pillars: symptomatic therapy, immunosuppressive therapy and, in the most advanced cases, renal replacement therapy [44].

### 4.1. Symptomatic Therapy

The main aim of symptomatic therapy in FSGS is to control the symptoms of the disease, namely, reducing intraglomerular pressure and persistent proteinuria, as well as preserving kidney function, without acting directly on the underlying cause. This approach is recommended for all forms of FSGS, with particular relevance in secondary FSGS, where patients tend not to respond to immunosuppression, making this option unsuitable [8].

Among the main symptomatic interventions are antihypertensive drugs, especially inhibitors of the renin-angiotensin-aldosterone system (RAAS), including angiotensin-converting enzyme inhibitors (ACEIs), such as ramipril, and angiotensin II receptor antagonists (ARAs), such as telmisartan. These drugs are considered first-line therapy for controlling blood pressure and proteinuria [8,34]. In addition, the administration of thiazide diuretics, such as hydrochlorothiazide and chlorthalidone—the latter has a longer duration of action and is more effective at excreting potassium—helps to control oedema and enhances the effect of RAAS inhibitors [8].

Lifestyle modification is also an essential component of symptomatic therapy. Stopping nicotine consumption is recommended, as smoking aggravates inflammation and accelerates the progression of glomerular damage, as well as contributing to an increase in blood pressure. Maintaining an adequate body weight is also essential, as excess weight imposes a hemodynamic burden on the kidneys, and is associated with hypertension and inflammatory processes that aggravate glomerular damage [22]. Dietary interventions, such as restricting the intake of sodium, potassium and calcium, also play an important role in controlling oedema and hypertension, while enhancing the effects of inhibiting RAAS [18,26]. Regular physical exercise, in turn, contributes to regulating blood pressure and improving general cardiovascular health [22].

Previous studies investigated the combination of angiotensin-converting enzyme inhibitors (ACEi) with angiotensin receptor blockers (ARBs) as a strategy to enhance proteinuria reduction. However, evidence from the ONTARGET trial and other meta-analyses demonstrated that this combination is associated with an increased risk of adverse cardiovascular events, hyperkalemia, and significant declines in glomerular filtration rate, particularly in elderly patients with cardiovascular comorbidities [48,49]. Consequently, current clinical guidelines recommend against combining ACEi and ARBs, favoring monotherapy with either agent to manage proteinuria and slow the progression of kidney disease [50,51].

An alternative therapeutic strategy involves combining an ACE inhibitor (ACEi) with an aldosterone antagonist, such as spironolactone, which, besides being a potassium-sparing diuretic, exhibits antifibrotic properties. This combination appears to have less impact on glomerular capillary pressure but carries a risk of hyperkalemia, particularly in patients with reduced glomerular filtration rate (GFR) [50,51]. This adverse effect can be mitigated by dietary restrictions and concomitant use of diuretics, although close clinical monitoring is essential [8].

### 4.2. Immunosuppressive Therapy

Currently, first-line therapy in primary FSGS is based on the use of immunosuppressive agents, which target the immune response and reduce inflammation, minimizing the progression of glomerular damage [12,32]. The main drugs used include glucocorticoids (GCs), such as prednisolone and methylprednisolone; calcineurin inhibitors (CNIs), such as cyclosporine A and tacrolimus; antiproliferative and antimetabolic agents, such as mycophenolate mofetil; monoclonal antibodies, such as basiliximab and rituximab; and alkylating agents, such as cyclophosphamide [12].

GCs and monoclonal antibodies stand out for their effectiveness in reducing proteinuria, and GCs are considered the first line of treatment in primary FSGS [22,44]. The choice of this approach is due to the association between immunosuppressant-induced remission and improved renal prognosis [22]. Although usually reserved for primary FSGS, immunosuppression can be considered in selected cases of secondary FSGS, especially in the presence of significant proteinuria [8].

Treatment with GC should be maintained for a minimum of four weeks until complete remission (CR) is achieved (Table 4) and is generally extended to a maximum of 24 weeks. However, prolonged use (up to 16 weeks) is associated with significant toxicity. After achieving CR, the dose should be maintained for a further two weeks before being gradually reduced. If only partial remission (PR) is achieved in the first eight weeks, therapy should continue with dose adjustment. In the absence of any therapeutic response (CR or PR) after eight weeks, or in the presence of adverse effects, it is recommended to switch to alternative therapies, such as NCIs [22].

In the table above, laboratory values are presented with their respective reference levels to ensure clarity. Proteinuria is expressed either as grams per day (g/day), reflecting total protein excretion measured in a 24 h urine collection, or as the urine protein-to-creatinine ratio (UPCR) in grams per gram creatinine (g/g creatinine), which estimates daily proteinuria from a spot urine sample. Serum albumin levels are reported in grams per deciliter (g/dL), with normalization considered when levels exceed 3.5 g/dL. These units allow for standardized assessment of renal function and remission status in patients with FSGS.

Complications associated with prolonged GC use include resistance (FSGS-RE), dependence (FSGS-DE) and frequent disease recurrence (FSGS-FR) (Table 5).

FSGS-RE is diagnosed in the absence of any response after 16 weeks of treatment. FSGS-DE occurs when there is a relapse during the gradual reduction in GCs or within two weeks of discontinuation. FSGS-RF is defined by the occurrence of two or more relapses in six months or more than four per year. A relapse is defined when proteinuria is greater than 3.5 g/day or 3.5 g/g creatinine after remission was previously achieved [22]. Prolonged therapy with GC is also associated with serious side effects, such as Diabetes *Mellitus*, cardiovascular disease and immunosuppression [2].

In cases where GCs are contraindicated, ineffective, or poorly tolerated, CNIs such as tacrolimus and cyclosporine A have emerged as therapeutic alternatives. Tacrolimus, in particular, has a lower incidence of adverse effects such as hirsutism and gingival hyperplasia, and is generally favored. Cyclosporine A is less effective in corticosteroid-resistant patients. Rituximab, an anti-CD20 monoclonal antibody, represents a second-line therapeutic option in cases of contraindication or intolerance to NCI, acting by depleting B cells and interrupting their interaction with T cells, helping to reduce proteinuria [26]. These options are also used in situations of FSGS-DE and FSGS-RE [22].

CNI should be administered for a minimum of four to six months before a lack of response is considered. If clinical remission occurs (CR or PR), therapy should be maintained for at least one year at the effective dose, followed by a gradual reduction over six to twelve months. Monitoring renal function is essential: increases of more than 30 percent in baseline creatinine justify reducing the dose, while a GFR of less than 30 mL/min/1.73 m^2^ requires reassessment of the continuity of treatment [22].

In cases refractory to pharmacological therapy, extracorporeal interventions can be considered, such as plasmapheresis, which aims to remove nephrotoxic circulating factors [32,52]; immunoadsorption, which removes specific antibodies from the plasma; and low-density lipoprotein apheresis (LDL-apheresis), which improves renal microcirculation, reduces proteinuria and podocyte damage [9].

The pharmaceutical and biotechnology industries are increasingly interested in developing new, safer and more effective therapeutic approaches for FSGS. Among the drugs currently under investigation are adalimumab (anti-TNF monoclonal antibody), pirfenidone (antifibrotic agent that inhibits TGF-β), fresolimumab (anti-TGF-β monoclonal antibody) and saquinavir (antiretroviral). However, these therapies have been associated with significant adverse effects, including acute kidney injury and serious infections, which have so far limited their routine clinical application [26].

The role of sodium-glucose cotransporter type 2 (SGLT2) inhibitors, such as dapagliflozin, in association with RAAS inhibitors, has also been studied in the treatment of proteinuric kidney disease in general. Although promising, more clinical data are needed to support their efficacy in FSGS. Another potential class is non-steroidal mineralocorticoid receptor antagonists, such as finerenone, which have demonstrated anti-inflammatory and anti-fibrotic properties [9].

The use of sirolimus, an mTOR inhibitor, has also been evaluated for the treatment of FSGS, but the available data indicate that it can accelerate the progression of the disease and is associated with significant toxicities; therefore, its use should be avoided. In addition, recent phase 3 clinical trials have shown efficacy in reducing proteinuria with new agents, including sparsentan, a molecule with a dual mechanism of action that antagonizes the angiotensin II receptor and the endothelin receptor (vasoconstrictor peptide), and abatacept, an immunosuppressant that blocks the CD80 pathway [8].

Table 6 summarizes the pharmacological treatment currently used in FSGS.

### 4.3. Renal Replacement Therapy

Despite therapeutic advances, many patients with FSGS continue to show unfavorable clinical progression, evolving into CKD or, in more advanced cases, end-stage renal failure. In these cases, renal replacement therapy becomes a vital support strategy and an option for prolonging survival. This approach includes hemodialysis, peritoneal dialysis and kidney transplantation. Dialysis allows excess fluids and toxins to be removed from the body, helping to normalize blood pressure and circulation. Most patients undergo hemodialysis sessions three times a week, which can allow them to maintain a functional life. However, the response to treatment is not uniform among all patients, and complications such as infections and malnutrition can significantly jeopardize the prognosis. It is noteworthy that infection continues to be one of the main causes of mortality among individuals on hemodialysis [44].

Kidney transplantation is the main option for cases of end-stage renal failure, with a significant impact on improving the quality of life and survival rate of these patients. However, the need for prolonged immunosuppression to prevent rejection of the transplanted organ entails additional risks, including opportunistic infections and adverse effects related to the drugs used. It is also important to consider that, even after a successful transplant, late rejection can occur, which jeopardizes the durability of the kidney graft [44].

## 5. Exploring Therapeutic Strategies for FSGS Using Cyclodextrins

Although the drugs currently used to treat FSGS have shown some therapeutic potential, they still have significant limitations, namely, low solubility in aqueous media, chemical instability, low targeting specificity and poor biocompatibility. These factors jeopardize the desired clinical efficacy [56].

In this context, it is imperative to develop new therapeutic strategies that offer better safety and efficacy profiles, with a view to inducing and maintaining remission of FSGS [56]. One of the most promising approaches is the use of advanced drug delivery systems, which allow for the controlled and targeted release of the active ingredient at the site of action, using innovative biotechnological technologies. These systems contribute to greater therapeutic efficiency, allowing lower doses of the drug to be used while reducing the incidence of adverse effects in patients.

**Figure 3:**
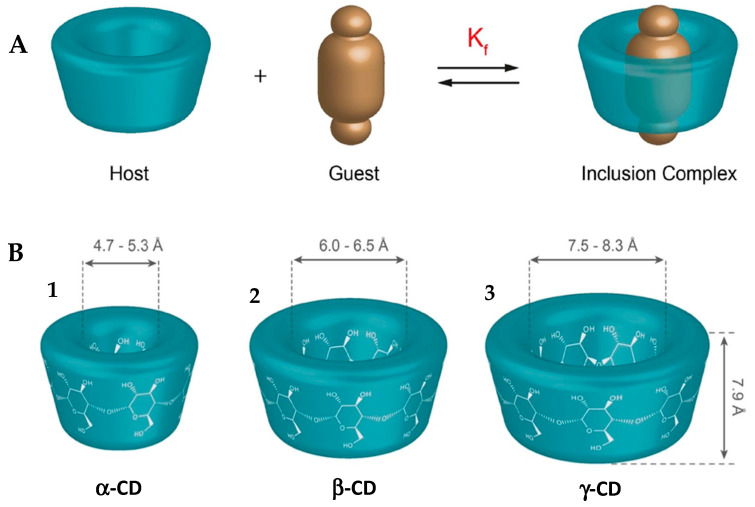
(**A**) Schematization of the formation of a complex through the inclusion of an organic molecule (the guest) in the CD (the host). (**B**) Schematic representation of the diameter of the inner cavity of the CDs. (**1**) α-Cyclodextrin; (**2**) β-Cyclodextrin; (**3**) γ-Cyclodextrin. Adapted from [63].

Among the most studied systems are those based on CDs, cyclic structures capable of forming inclusion complexes with various molecules, particularly drugs (Figure 3A) [56,57].

The use of CDs has proved effective in improving the aqueous solubility of hydrophobic compounds, stabilizing nanoparticles, promoting cellular absorption and more selective delivery of drugs to the target tissue [58]. These effects are especially relevant in the context of FSGS, given the need for highly targeted therapeutic interventions [56].

Cyclodextrins are considered in GRAS (Generally Recognized as Safe) parameters and practically non-toxic after oral administration. However, there are reports that treatment with β-CD significantly increased urea and creatinine levels in rats, parameters that are associated with liver damage [59]. In high doses, cyclodextrins can cause diarrhea, nausea and vomiting as adverse effects, but these are extremely rare [60].

Of the three main natural CDs—α, β and γ—the β-CD stands out as the most widely used in the pharmaceutical industry. This preference is largely due to the ideal size of its hydrophobic cavity, which adapts to the majority of drugs in clinical use (Figure 3B). In addition, β-CD has a proven ability to increase the bioavailability of drugs and reduce their toxicity. Its additional advantages include ease of production and low costs [28,61,62].

It is also worth noting that CDs—α, β and γ—have the ability to bind to cholesterol, promoting a reduction in its accumulation in tissues [62,63]. This property is particularly relevant in the treatment of FSGS, since lipid deposition in the kidneys has been implicated in the pathogenesis and progression of the disease, with podocytes being especially vulnerable to the damaging effects induced by lipids [9].

Recent studies have explored the efficacy of combining CDs with drugs used in the treatment of FSGS, with promising results in terms of improving therapeutic efficacy and reducing adverse effects. The main physicochemical characteristics of these CDs are summarized in Table 7.

**Figure 4:**
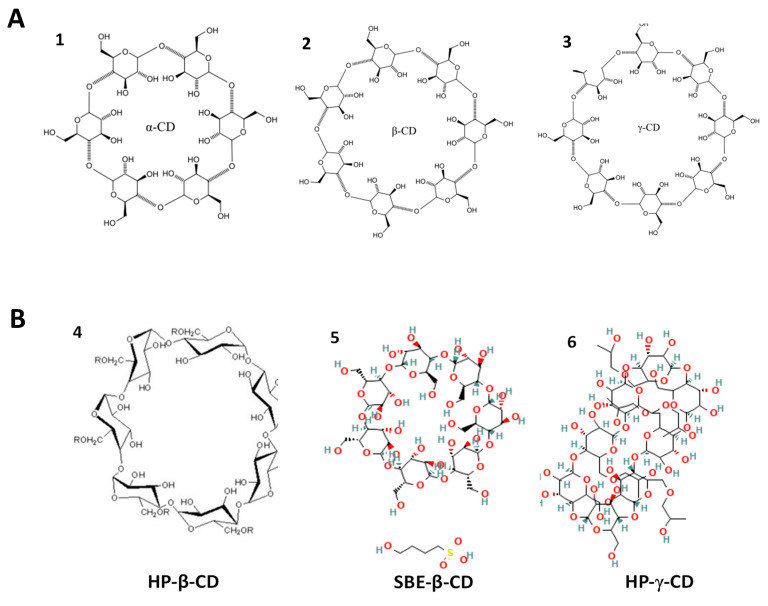
(**A**) Schematic representation of the chemical structures of natural cyclodextrins: (**1**) α-CD, (**2**) β-CD, and (**3**) γ-CD. (**B**) Schematic representation of the structures of the modified cyclodextrins: (**4**) hydroxypropyl-β-CD (HP-β-CD), (**5**) sulfobutylether-β-CD (SBE-β-CD), and (**6**) hydroxypropyl-γ-CD (HP-γ-CD). Adapted from [67,68,69,70,71,72].

In addition to natural CDs, chemically modified CDs have also been investigated, obtained by functionalizing the hydroxyl groups of the original molecule, with the aim of improving the solubility and versatility of these structures in pharmaceutical formulations (Figure 4) [61,65,66].

The following sections present a detailed analysis of the main natural CDs—α, β and γ—with a focus on their potential to increase the efficacy and safety of drugs used in the treatment of FSGS.

### 5.1. Cyclodextrins as Drug Transport Vehicles

#### 5.1.1. Alpha Cyclodextrin (α-CD)

α-CD has been investigated for its favorable physicochemical properties in the context of drug delivery systems, especially its high solubility in aqueous media, which is higher than that observed for β-CD [20]. However, the application of α-CD is limited by its smaller cavity diameter, which restricts its ability to form a complex with smaller molecules [65].

Although no specific studies have been carried out on α-CD in models of FSGS, there is preclinical evidence to support its potential as a vehicle for drugs used in the treatment of this disease. One relevant example is the study carried out by Jóhannsdóttir et al. which evaluated the ability of CDs—α, β and γ—to increase the aqueous solubility of cyclosporine A, an immunosuppressant widely used in the treatment of FSGS [73]. Cyclosporine A has reduced aqueous solubility, attributed to its high lipophilicity, which jeopardizes its oral bioavailability and stability in liquid formulations. The results obtained in this study showed that all the CDs tested promoted a significant increase in the solubility of cyclosporine A in aqueous media, especially α-CD, which had the greatest solubilizing effect (Table 8). This result suggests that cyclosporine A has a greater affinity for CDs with smaller diameter cavities, compatible with the structure of α-CD. In addition, the formation of inclusion complexes with CDs contributed to the stabilization of the cyclosporine A molecule, preventing its degradation in aqueous media. The authors confirmed that the encapsulation of the lipid portions of cyclosporine A inside the cavity of the CDs was responsible for improving their physicochemical properties.

It should be noted that the tests carried out, namely, the development of aqueous eye drops containing the cyclosporine A/CD complex, were conducted on animal models not specifically related to FSGS [73].

#### 5.1.2. Beta Cyclodextrin (β-CD)

**Figure 5:**
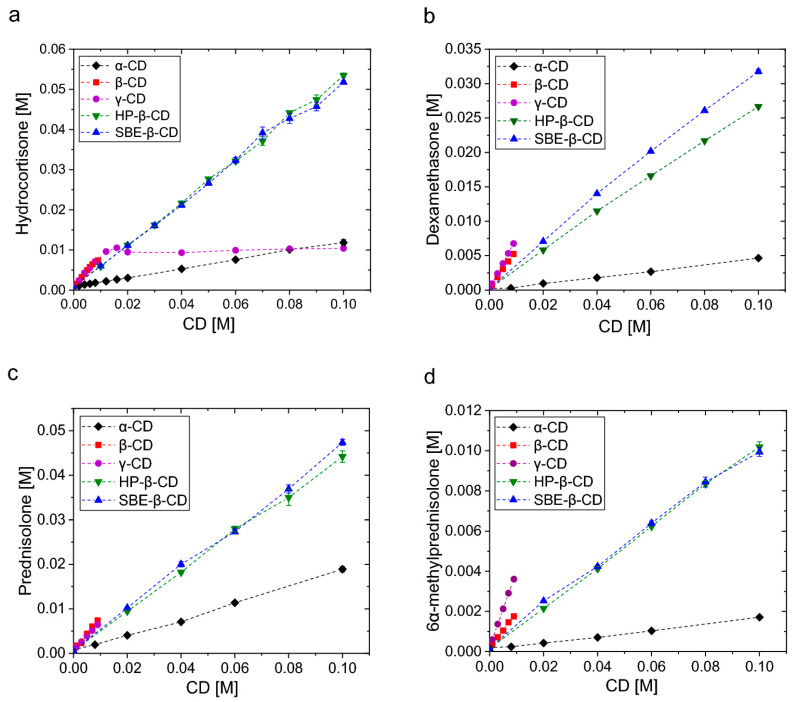
Phase solubility diagrams obtained for (**a**) hydrocortisone, (**b**) dexamethasone, (**c**) prednisolone and (**d**) 6α-methylprednisolone, in solutions containing α-, β-, γ-cyclodextrin, HP-β-CD or SBE-β-CD, at 23 °C. Results are presented as mean ± standard deviation (*n* = 4). Adapted from [77].

Among the different types of CDs, β-CD and its derivative forms have been the most extensively studied in the context of drug delivery systems. One of the most relevant derivatives is 2-HP-β-CD (Figure 5), widely recognized for its safety and approved for use in various routes of administration, including parenteral, oral, rectal, transdermal and ophthalmic [74].

2-HP-β-CD has attracted particular interest for its therapeutic potential in glomerular diseases, including FSGS, due to its ability to remove or redistribute cholesterol from tissues, a factor implicated in the progression of the disease. Recent evidence shows that dysregulation of cholesterol homeostasis, including alterations in the expression of regulatory genes, is associated with greater susceptibility to FSGS and other lipotoxic nephropathies [9,63]. In addition, several studies have explored the use of this CD as a delivery system for drugs used in FSGS, although they have not been carried out specifically in models of this disease. Cirri et al. demonstrated that incorporating hydrochlorothiazide into 2-HP-β-CD significantly increased its aqueous solubility and dissolution rate, promoting a more controlled release, an aspect that is especially relevant in pediatric formulations [75].

Spironolactone, used as an adjuvant drug in FSGS due to its aldosterone antagonist action, has been the target of pharmaceutical reformulation strategies. In the study by Lopalco et al., an oral solution of spironolactone complexed with 2-HP-β-CD was developed, allowing for a significant increase in its solubility in aqueous media. This approach demonstrates the applicability of CDs in modulating the biopharmaceutical properties of hydrophobic drugs, reinforcing their potential in specific clinical contexts such as FSGS [76].

In fact, recent studies have reinforced the potential of CDs as promoters of the solubilization and bioavailability of drugs with low water solubility. Fereidounpour et al. evaluated the formation of complexes between different CDs (α, β, γ and their derivatives) and steroids widely used in clinical settings, such as hydrocortisone, dexamethasone, prednisolone (the latter being widely used in the treatment of FSGS) and 6α-methylprednisolone. The results showed a significant improvement of the drugs with increasing CD concentration, especially with modified β-CDs, such as hydroxypropyl-β-CD (HP-β-CD) and sulfobutylether-β-CD (SBE-β-CD). In addition to the experimental solubility tests, the authors applied computational modeling (molecular docking and MM/GBSA), the results of which showed a strong correlation with the empirical data (R^2^ 0,94), validating the predictive model (Figure 5). Although this study was not conducted in the context of FSGS, the steroids in question are used in the treatment of this pathology, and the results obtained provide a solid basis for extrapolating the use of CDs, particularly β-CDs derived from them, as vehicles for their administration in this disease. The evidence that these CDs not only improve solubility but also stabilize the interaction with drugs reinforces the proposal for their use in FSGS as a complementary therapeutic strategy that could be explored in future experimental trials [77].

**Figure 6:**
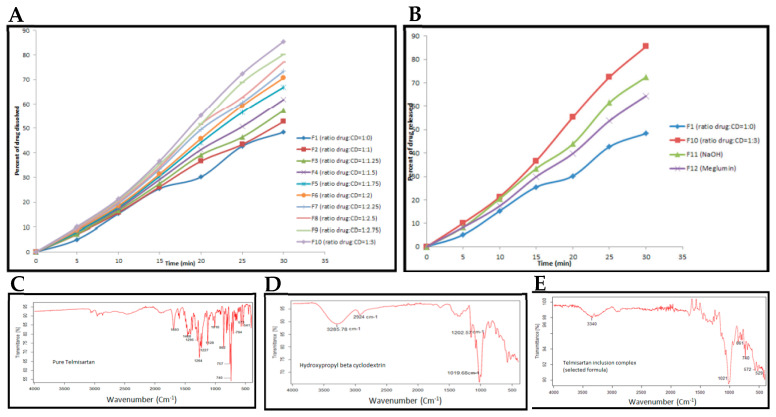
(**A**) Dissolution profiles of formulations F1 to F10 containing telmisartan complexed with HP-β-cyclodextrin. (**B**) Comparison of the dissolution profiles of formulation F10 with formulations containing NaOH, meglumine and pure telmisartan. Fourier transform infrared (FTIR) spectra of pure telmisartan (**C**), HP-β-cyclodextrin (**D**), and the inclusion complex (1:3) (**E**). Adapted from [78].

In a study conducted by Hasson and Ghareeb, a formulation of telmisartan complexed with 2-HP-β-CD was developed in order to improve its aqueous solubility. Various proportions were tested, with the F10 formulation (1:3, drug:CD) obtaining the best results, with a dissolution percentage of 99.6 percent in 45 min, compared to just 26.5 percent for the pure telmisartan formulation. DSC, FTIR and PXRD analyses confirmed the formation of the complex and the transition to an amorphous state, justifying the increase in solubility. In addition, the tablets showed physicochemical stability for three years, maintaining the dissolution profile. This evidence reinforces the value of CDs, particularly 2-HP-β-CD, as a technology applicable to drugs with similar challenges, also used in FSGS. Figure 6A shows a progressive increase in the dissolution percentage with the increase in the drug:CD ratio, reaching a maximum value of approximately 99.6% in the F10 (1:3) formulation, in contrast to 26.5% for pure telmisartan (F1), demonstrating the enhancing effect of cyclodextrin on solubility. The F10 formulation showed the best dissolution performance, outperforming conventional strategies with solubilizing agents such as NaOH and meglumine, which shows the effectiveness of complexation with HP-β-CD (Figure 6B). The alteration and/or disappearance of bands characteristic of telmisartan in the spectrum of the complex indicate intermolecular interactions between the drug and the cyclodextrin, confirming the formation of the inclusion complex without the occurrence of chemical degradation (Figure 6C–E) [78].

**Figure 7:**
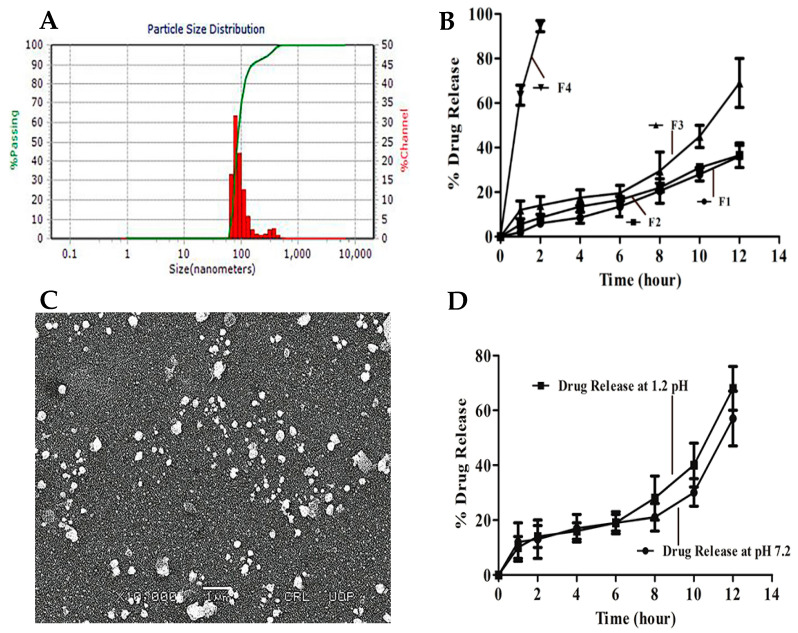
(**A**) Particle size distribution and polydispersity index (PDI) of solid lipid nanoparticles (SLNs) containing mycophenolate mofetil. (**B**) Scanning electron microscopy (SEM) images of SLNs loaded with mycophenolate mofetil. (**C**) In vitro release profile of mycophenolate mofetil from SLNs over 12 h. (**D**) Comparison of the drug release profile in different media (pH 1.2 e 7.2). Adapted from [79].

Iqbal et al. conducted a study in which they incorporated mycophenolate mofetil (MMF), one of the immunosuppressive drugs used in the conventional treatment of FSGS, into solid lipid nanoparticles (SLNs) using the nanoprecipitation method, with the aid of β-CD. Although the study does not detail inclusion complexation, β-CD played a facilitating role in the formulation process, favoring encapsulation efficiency and particle stability. The analyses revealed particles with an average size between 80 and 170 nm and a low polydispersity index (less than 0.3), indicating a relatively uniform size distribution suitable for oral therapeutic applications, allowing for a stable and homogeneous formulation to be obtained (Figure 7A). Scanning electron micrography (Figure 7B) confirmed a spherical morphology, smooth surface and size consistent with the data obtained by dynamic light scattering (DLS), confirming the integrity and homogeneity of the formulation, typical of well-structured SLNs. The in vitro release profile showed sustained behavior over 12 h (Figure 7C), with approximately 68% of the drug released in acidic media (pH 1.2) and 56% in neutral media (pH 7.2), as illustrated in Figure 7D, suggesting that the lipid matrix favors intestinal release, a desirable condition for improving oral absorption. These release rates indicate controlled kinetics with potential application in once-daily dose regimes. In addition, the different formulations tested (F1 to F4), shown in Figure 7B, showed variations in release rate depending on composition, allowing for specific optimizations of the lipid matrix, which contrasts with the faster release of non-nanostructured forms. These results suggest feasibility for a once-daily administration regime [79].

**Figure 8:**
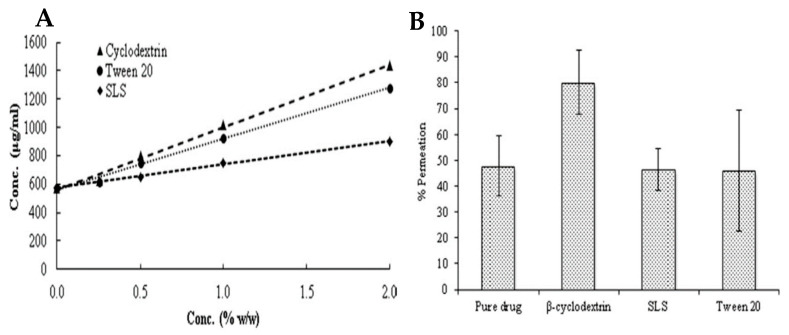
(**A**) Solubility of hydrochlorothiazide in different concentrations of β-CD, Tween 20 and sodium lauryl sulphate (SLS). (**B**) Percentage of permeability of the drug without inclusion in any solubilizing agent, in β-CD, in Tween 20, and SLS. Adapted from [80].

Altamimi et al. investigated the increase in solubility, stability and permeability of hydrochlorothiazide by including this drug in β-CD, which was used as a solubilizing agent. With regard to the aqueous solubility of the drug, at the end of the study, there was a linear increase when the drug was included in β-CD, and it was found that, compared to other solubilizing agents, this CD increased the solubility of the drug the most (Figure 8A). In addition, permeability was assessed in an ex vivo test, which revealed that the fraction of hydrochlorothiazide absorbed when using β-CD increased significantly and that, as was observed with solubility, β-CD was also found to promote a more marked increase in drug permeability compared to other solubilizing agents (Figure 8B). In terms of drug stability, it was found that β-CD was able to preserve the chemical integrity of the compound, without altering its physicochemical properties, for a period of 60 days at room temperature [80].

**Figure 9:**
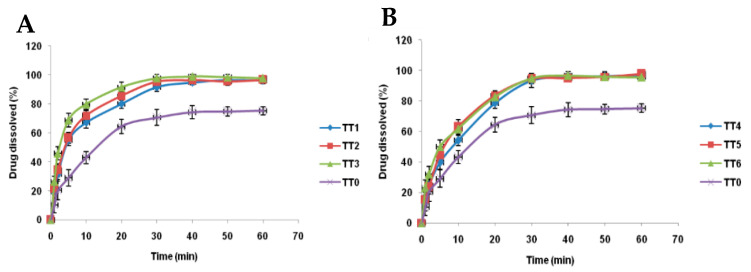
(**A**) In vitro drug dissolution studies on sublingual tablets (TT0 and TT1-TT3). (**B**) In vitro drug dissolution studies on sublingual tablets (TT0 and TT4-TT6). Data are presented as mean values with error bars corresponding to the standard deviation (n = 3). Adapted from [81].

In addition, Mohanan et al. conducted a study that consisted of evaluating different formulations of tacrolimus sublingual tablets containing β-CD, with the aim of improving the solubility and dissolution rate of the drug. All the formulations tested had the same molar ratio of 1:2 (drug: β-CD), the variable being the method of preparing the inclusion complex. The results showed that all the formulations with β-CD promoted a significant improvement *in* in vitro dissolution compared to tacrolimus alone (Table 9). Among these, the TT3 formulation, prepared by kneading, stood out as having the highest dissolution rate, which indicates more efficient complexation and an increased potential for increasing the bioavailability of the drug (Figure 9) [81].

#### 5.1.3. Gamma Cyclodextrin (γ-CD)

As observed with α-CD, γ-CD has a higher aqueous solubility than β-CD [20], which makes it a promising candidate for pharmaceutical formulations aimed at improving the bioavailability of hydrophobic compounds. However, in contrast to α-CD, γ-CD has an internal cavity with a larger diameter, allowing it to form inclusion complexes with larger molecules, expanding its potential for use in transporting larger drugs [65].

Although there are no studies to date that have directly evaluated the effects of γ-CD in experimental models of FSGS, several studies have demonstrated its technological advantages with drugs of interest for the treatment of this pathology.

Bíró et al. developed an inclusion system between prednisolone (PR) and 2-HP-β-CD (HP-β-CD) and hydroxypropyl-γ-CD (HP-γ-CD), with the aim of increasing the efficacy of ocular administration of this corticosteroid. The results showed that the solubility of PR increases linearly with the increase in concentration of both CDs tested. This linear behavior is characterized as a Higuchi AL diagram, indicating the formation of inclusion complexes in a 1:1 molar ratio between the drug and the CDs. Analysis of the apparent stability constants of the complexes revealed that PR has a greater affinity for HP-γ-CD than for HP-β-CD. This result suggests that the PR-HP-γ-CD complex is thermodynamically more stable. However, the slope of the regression line obtained from the solubility curve was higher for HP-β-CD, indicating that it has a higher solubilization capacity compared to HP-γ-CD, even with a slightly lower stability constant. As is well known, the linear regression equation makes it possible to determine the concentration of each CD needed to solubilize the desired amount of active ingredient, which is essential for defining the ideal formulation conditions and avoiding the excessive use of excipients. Therefore, although HP-γ-CD forms more stable complexes with PR, HP-β-CD has been shown to be more effective in terms of solubilization and can therefore be considered the best option for developing water-based formulations containing PR [82].

Muankaew et al. investigated the impact of γ-CD on the permeability of lipophilic and poorly permeable drugs, namely, hydrochlorothiazide, irbesartan and telmisartan, all of which have clinical applications in the symptomatic treatment of FSGS. Using a diffusion cell system with fused membranes, the authors showed that γ-CD increased the permeability of all the drugs tested. However, the degree of increase was variable and dependent on the specific physicochemical properties of each compound. This effect is particularly relevant since increased permeability can reduce the therapeutic doses required, helping to minimize the associated adverse effects [83].

**Figure 10:**
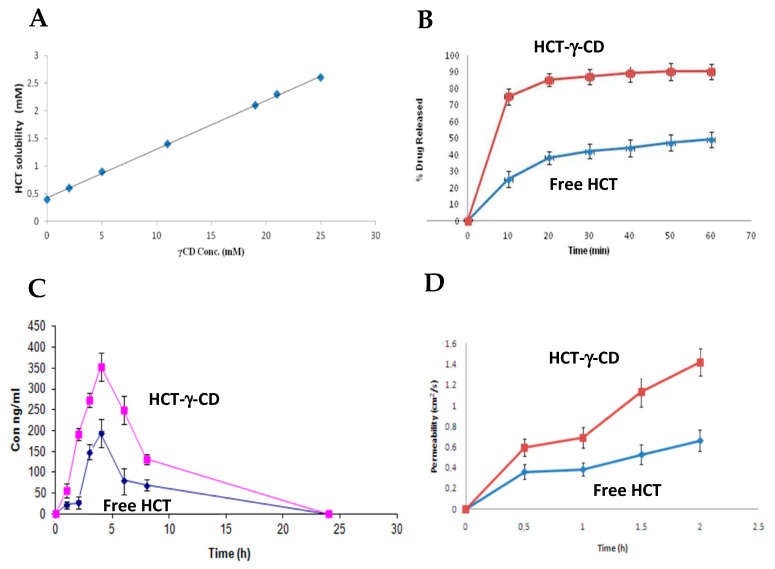
(**A**) Phase solubility diagram of hydrochlorothiazide (HCT) in the presence of γ-cyclodextrin (γ-CD). (**B**) Dissolution profiles of HCT in free form and the form of an inclusion complex with γ-cyclodextrin (HCT-γ-CD). (**C**) Mean plasma concentration profiles as a function of time (±standard deviation) of hydrochlorothiazide (HCT) and the HCT-γ-CD inclusion complex, after oral administration of 25 mg in beagle dogs (*n* = 5). (**D**) Intestinal permeability as a function of time, assessed using the Ussing chamber, for HCT and the HCT-γ-CD inclusion complex. Adapted from [84].

Along the same lines as the study described above, Masmoudi, S. et al. investigated the effect of γ-CD on the aqueous solubility, dissolution rate, oral bioavailability and intestinal permeability of hydrochlorothiazide (HCT). The inclusion complex was prepared by the co-precipitation method, by dissolving γ-CD and hydrochlorothiazide in a hydroalcoholic medium (50:50, *v/v*), under agitation for 72 h at 40 °C. The results showed a significant increase in the aqueous solubility of HCT as a function of the concentration of γ-CD, as shown in Figure 10A [84].

The dissolution rate was assessed in vitro in an acidic medium (0.1 N HCL) and there was a marked improvement in the dissolution profile of the HCT-γ-CD inclusion complex compared to the free form of HCT. The complex showed a substantially higher dissolution rate, with around 75 percent of the drug dissolved at 10 min, compared to 20 percent for free HCT. After 60 min, the complex reached approximately 90% dissolution (Figure 10B) [84].

Based on these results, an in vivo study was carried out in dogs (*n* = 5). The HCT-γ-CD complex showed significantly higher bioavailability than free HCT, reflected by an increase in Cmax (from 192.85 ± 6.31 ng/mL to 353.22 ± 33.84 ng/mL; *p* < 0.05) and AUC (from 1268.2 ± 102.6 to 2746.3 ± 164.8 ng/mL; *p* < 0.05), as illustrated in Figure 10C [84].

Finally, intestinal permeability was assessed ex vivo using segments of jejunum mounted in a Ussing chamber. The results revealed a significant increase in the permeability of the HCT-γ-CD complex compared to the free drug, with the apparent permeability coefficients (Papp) rising from 1.96 ± 0.27 × 10^−6^ cm/s to 3.93 ± 0.81 × 10^−6^ cm/s (*p* < 0.05), as represented in Figure 10D [84].

In a complementary way, Thi et al. investigated the formation of inclusion complexes between methylprednisolone and three different types of CDs: γ-cyclodextrin (γ-CD), 2-hydroxypropyl-γ-cyclodextrin (HP-γ-CD) and 2-hydroxypropyl-β-cyclodextrin (HP-β-CD), with the aim of evaluating their effects on the aqueous solubility of the drug. By analyzing phase solubility diagrams, the authors showed that the solubility of methylprednisolone increased significantly as the concentration of CDs increased. Both HP-β-CD and HP-γ-CD showed solubility profiles of the A_L_ type, characterized by a linear increase in solubility as a function of CD concentration, indicating the formation of soluble inclusion complexes in aqueous media. In the case of γ-CD, a B_S_ profile was observed, i.e., increasing solubility up to a certain concentration (~0.03 M), followed by precipitation. This behavior shows that the complexes formed between methylprednisolone and γ-CD have limited solubility in aqueous media. The order of affinity for the formation of the complexes (based on the apparent stability constants was γ-CD > HP-γ-CD > HP-β-CD, suggesting that the larger cavity of γ-CD favors more effective interactions with methylprednisolone, while the hydroxypropyl groups present in the derivative CDs may induce steric hindrance to complexation [85].

Confirmation of the formation of the inclusion complexes was obtained by ^1^H-NMR spectroscopy, which showed significant chemical shifts in the internal protons of the cavity of the CDs and in the protons of methylprednisolone, especially in the A and B rings, suggesting its entry into the cavity of γ-CD and HP-γ-CD. In the case of HP-β-CD, inclusion seems to occur through the C and D rings, although the results also support a possible deep insertion of the A and B rings. The experimental data were further supported by molecular modeling, which confirmed the more stable geometry of the complex with γ-CD, with methylprednisolone penetrating through the wider end of the CD cavity. Although this study was not carried out on specific models of FSGS, the results obtained indicate that γ-CD and its modified forms, by increasing the aqueous solubility of hydrophobic drugs such as methylprednisolone, may represent a promising strategy for optimizing the formulation and oral bioavailability of these agents in the context of the treatment of this pathology. Further studies in specific models of FSGS are therefore warranted to explore the therapeutic impact of these γ-CD-based inclusion systems [85].

Table 10 summarizes the main results obtained in experimental studies with CDs and drugs used to treat FSGS.

### 5.2. Cyclodextrins as Therapeutic Agents

#### 5.2.1. Alpha Cyclodextrin (α-CD)

Several studies have shown that, in addition to their traditional role as pharmaceutical excipients, CDs have relevant biological properties that justify their consideration as active therapeutic agents. α-CD, in particular, has stood out for its beneficial effect on key metabolic parameters, with a potential impact on the management of systemic diseases. In a randomized, double-blind, placebo-controlled clinical trial conducted by Amar et al., oral supplementation with α-CD (6 g/day for 12 to 14 weeks) resulted in a statistically significant reduction in LDL particles (−10%), as well as improvements in fasting blood glucose levels and insulin resistance (HOMA-IR). These effects were observed in healthy adults, reinforcing its therapeutic potential even outside classic risk populations. This profile is particularly relevant in the context of FSGS, a pathology associated with lipid alterations, inflammation and oxidative stress, areas in which α-CD could exert a beneficial modulating action [86].

It is important to note that, to date, no clinical trials have been published on the specific use of α-CD in patients with kidney disease. The clinical data available only refers to the fact that α-CD significantly improves metabolic parameters, such as cholesterol levels, as mentioned in the study described above. It is therefore important that these studies be promoted in view of the evidence that this CD has beneficial effects in reducing high cholesterol levels, particularly LDL cholesterol, parameters directly associated with the development of kidney disease.

#### 5.2.2. Beta Cyclodextrin (β-CD)

Mitrofanova et al., carried out a study with the aim of directly investigating the effects of 2-HP-β-CD in experimental models of FSGS and Alport syndrome. In this study, BALB/c mice were subjected to adriamycin-induced nephropathy, a model widely used to mimic FSGS. After inducing kidney damage, the animals were treated subcutaneously with 2-HP-β-CD for 10 weeks. The results revealed a significant reduction in the albumin-to-creatinine ratio and urea levels, accompanied by a decrease in mesangial expansion, i.e., an increase in the glomerular cells of the kidney, observed in histological analyses; however, serum creatinine remained unchanged. These results suggest that 2-HP-β-CD, administered alone, has a direct protective effect on the kidney, attenuating proteinuria and glomerular damage, possibly by modulating tissue lipid content [87].

**Figure 11:**
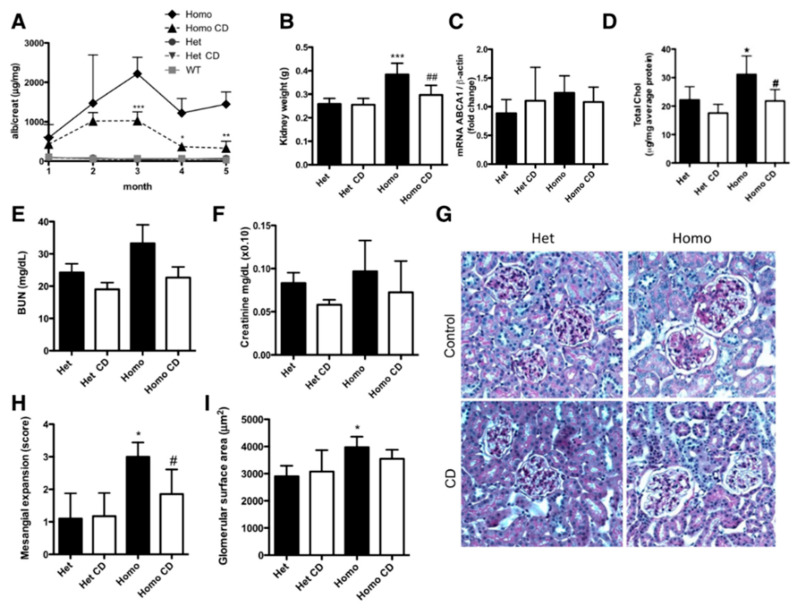
Graphical representation of the action of 2-HP-β-CD on the progression of diabetic kidney disease in vivo. (**A**) Administration of 2-HP-β-CD in homozygous and heterozygous mice, which resulted in a reduction in albumin−creatinine levels. (**B**) Decrease in kidney weight of mice treated with 2-HP-β-CD. (**C**) Effect of 2-HP-β-CD on ABCA1 mRNA expression in the renal cortex of homozygous and heterozygous mice. (**D**) Effect of 2-HP-β-CD on total cholesterol levels in the kidney of rats. (**E**,**F**) Serum concentrations of blood urea nitrogen and creatinine in the kidney of rats after treatment with 2-HP-β-CD. (**G**) Representative staining of sections of the kidney of rats after five months of treatment with 2-HP-β-CD compared with the group not treated with 2-HP-β-CD. Bar graph representation (mean ± SD) showing the scores for mesangial expansion (**H**) and glomerular surface area (**I**) in PAS-stained kidney sections from homozygous and heterozygous BTBR *ob/ob* mice after 5 months of treatment with either CD or vehicle. The data were evaluated by two independent, blinded investigators. * *p* < 0.05, ** *p* < 0.01, *** *p* < 0.001 when comparing DKD+ to C. # *p* < 0.05, ## *p* < 0.01 when comparing CD-treated mice to untreated ones within the same group. Het: heterozygous; Homo: homozygous; WT: wild type. Adapted from [88].

Additionally, Merscher-Gomez et al. demonstrated, both in vivo (human podocyte cells exposed to serum from diabetics with albuminuria) and in vivo (BTBT ob/ob murine model of diabetes, recognized as a robust model of progressive diabetic nephropathy), that the induction of cholesterol efflux mediated by HP-β-CD preserves podocyte integrity and reduces markers of kidney damage. In the animal model, subcutaneous administration of CD resulted in a significant reduction in albuminuria, mesangial expansion, renal lipids and renal weight, as well as an improvement in metabolic parameters (fasting glycemia, glucose tolerance and insulinemia). These results not only highlight the role of HP-β-CD as a modulator of lipid homeostasis, but also point to a direct action in protecting the glomerular filtration barrier, an effect that may be relevant in the pathophysiology and management of FSGS. The ability of HP-β-CD to promote the removal of intracellular cholesterol and mitigate cell damage makes it a promising candidate as an adjuvant or alternative therapy in chronic kidney diseases associated with dyslipidemia and podocyte injury. Specifically, Merscher-Gomez et al. demonstrated that subcutaneous administration of HP-β-CD (4000 mg/Kg, three times a week) for five months resulted in a significant reduction in the albumin-to-creatinine ratio from the third month of treatment, with this effect being maintained until the animals were sacrificed (Figure 11A). This functional improvement was accompanied by a reduction in kidney weight (Figure 11B) and a significant decrease in total kidney cholesterol (Figure 11D), although there were no significant changes in the expression of ABCA1 (expression of the ATP transporter mRNA) in the kidney cortex (Figure 11C). Although plasma urea nitrogen and creatinine values were not significantly modified (Figure 11E,F), a reduction in glomerular mesangial expansion was observed (Figure 11G,H), with glomerular area remaining unchanged (Figure 11I). These results reinforce the hypothesis that HP-β-CD has beneficial effects on preserving glomerular morphology and function, possibly by removing cholesterol and modulating the intrarenal lipid environment, in the same way that it reduces albumin−creatinine levels, contributing to the protection of podocytes and their respective function, preventing the progression of diabetic kidney disease, which could be highly relevant for glomerular diseases such as FSGS, in which podocyte dysfunction and lipid accumulation contribute significantly to the progression of the disease [88].

## 6. Final Considerations and Future Prospects

Focal segmental glomerulosclerosis is a progressive kidney disease with an increasing prevalence worldwide, for which there is still no proven curative therapy. The drugs currently used are mainly aimed at delaying the progression of the disease, but their clinical efficacy is limited by factors such as low solubility in aqueous media, chemical instability, reduced availability and lack of selectivity for renal tissue.

In this context, advanced drug delivery systems, particularly those based on cyclodextrins, are proving to be promising strategies. CDs, particularly the variants α, β and γ, have demonstrated their ability to form inclusion complexes with hydrophobic compounds, improving crucial pharmacokinetic parameters such as solubility, stability and bioavailability. Studies already carried out with drugs used in FSGS, but applied in other pathological models, indicate that their incorporation into CDs significantly enhances the therapeutic efficacy of these compounds, which supports the relevance of exploring this approach specifically in FSGS.

In addition, relevant evidence is beginning to emerge that CDs can show intrinsic therapeutic activity, independently of the incorporation of drugs. Results obtained with different derivatives, in particular 2-hydroxypropyl-β-cyclodextrin (2-HP-β-CD), have shown direct beneficial effects on kidney function and podocyte integrity in kidney disease models, namely, by reducing proteinuria, decreasing cholesterol accumulation in the kidneys and modulating the inflammatory response. In addition, α-cyclodextrin has shown positive metabolic effects in humans, particularly in regulating lipid and glycemic profiles, which may indirectly influence the clinical progression of FSGS [87].

It should be emphasized, however, that the therapeutic use of CDs, particularly β-CD, is not without risks. Its high affinity for cholesterol can lead to the formation of poorly soluble complexes with nephrotoxic potential, which justifies the need for detailed safety studies [20,28]. In addition, although the most robust results to date have been obtained with β-CD and its derivatives, it will be essential to conduct research with other CDs, such as α-CD and γ-CD, in the specific context of FSGS. This is because the efficacy of CDs can vary significantly depending on the drug in question, the type of target tissue and the desired pharmacological profile, making the choice of CD a critical factor that should not be seen as indifferent.

The scarcity of direct experimental studies testing the efficacy of CDs in FSGS is a major limitation in the clinical translation of this evidence. This work, therefore, seeks to contribute to a paradigm shift in the therapeutic approach to FSGS, pointing to CDs not just as functional excipients but as potential active therapeutic agents with a direct impact on the modulation of relevant pathological pathways. In view of the results already available, it is considered a priority to carry out preclinical and clinical trials specifically focused on FSGS, to validate the efficacy and safety of these strategies, as well as to deepen their interaction with the pathophysiological mechanisms of the disease.

At the same time, identifying specific biomarkers of therapeutic response remains an unsolved challenge [89]. The use of tools capable of assessing the efficacy of treatments, particularly glucocorticoids, could enable a more personalized approach, minimizing adverse effects and delaying progression to end-stage renal disease [8,26].

In short, the use of CDs is an innovative approach with dual therapeutic potential, as drug vehicles and as active compounds, which merits in-depth and targeted research in the context of FSGS. Future translation into clinical trials and the discovery of new biomarkers are priority objectives for the advancement of personalized therapeutics for FSGS.

## Figures and Tables

**Table 1:** Main genes and proteins related to the development of FSGS. Adapted from [10].

Gene	Protein
***NPHS1***	Nephrin
***NPHS2***	Podocin
***PLC 1ε***	Phospholipase C 1ε
***WT1***	Wilms tumor 1
***LAM 2β***	Laminin 2β
***PTP-RO***	Protein tyrosine phosphatase receptor type O
***ARHGDIA***	Rho GDP dissociation inhibitor α
***ADCK4***	AarF domain containing kinase 4
***EMP2***	Epithelial membrane protein 2
***ACTN4***	α-Actinin-4
***TRPC6***	Transient receptor potential cation channel 6
***CD2AP***	CD2-associated protein
***APOL1***	Apolipoprotein L1
***INF2***	Inverted formin-2
***MYO1E***	Myosin 1E
***PAX2***	Paired box gene 2
***ANLN***	Anilin
***CRB2***	Crumbs homolog 2

**Table 2:** Diagnostic methods for FSGS.

Diagnostic Methods		Description	References
**Blood tests**	Serum Creatinine and Glomerular Filtration Rate (GFR)	Assesses renal function; Creatinine may be normal early in the disease, but may increase as FSGS progresses to renal failure.	[32]
	Serum albumin	It is usually reduced in patients with nephrotic syndrome associated with FSGS.	
	Circulating biomarkers	**SuPAR** (Soluble urokinase-type Plasminogen Activator Receptor) is a soluble receptor that, when at high levels, is associated with podocyte injury; It is the most relevant biomarker for FSGS and has strong diagnostic and prognostic potential.	[8]
		**CD80** (Cluster of Differentiation 80) is a protein that helps differentiate FSGS from other podocyte pathologies, for example, Minimal Change Disease (MCD).	
**Urine tests**	Urinary protein/ Creatinine ratio	Assesses proteinuria (>3.5 g/day)	[32]
	Urinary biomarkers	Detects urinary podocin and nephrin, which indicate podocyte damage, and MCP-1 (Monocyte Chemoattractant Protein-1), which is a urinary inflammatory marker.	[10]
**Imaging exams**	Helps rule out other causes of kidney disease. The kidneys may be normal in size in the early stages, but may lead to atrophy in later stages.		[8]
**Kidney biopsy**	Optical microscopy	It detects glomerular size, microcystic tubular changes, tubular hypertrophy and morphological variants of FSGS.	[8]
	Immunofluorescence microscopy	It rules out other primary glomerulopathies.	
	Electron microscopy	It reveals fusion of pedicels, cytoplasmic projections of podocytes, characteristics of FSGS, microvillous transformation of podocytes, tubuloreticular inclusions; It differentiates FSGS from MCD (it has diffused fusion without glomerular sclerosis).	
**Genetic testing**	It allows for more appropriate therapy for patients, for example, avoiding glucocorticoids (except in genetic forms that may be responsive), a better prognosis (typical results of the native kidney and the likelihood of resorting to a kidney transplant) and identification of family history (identification of the disease in other family members and prenatal testing); Recommended when there is a family history to identify gene mutations associated with autosomal dominant mutations, such as Interferon (IFN) and α-Actinin-4 (ACTN4).		[8]

**Table 3:** Morphological variants, characterization and relationship with the type of FSGS. Adapted from [8].

Morphological Variant	Characterization	Type of FSGS
**Not Otherwise Specified—NOS**	The most common form, progressive podocyte injury	Primary
**Collapsing**	Aggressive form, with glomerular collapse and proliferation of epithelial cells. Associated with HIV, viral infections and genetic mutations	Primary; Virus-mediated; Associated with pharmaceuticals; Associated with APOL1
**Perihilar**	Characterized by sclerosis predominantly around the hilum (point of entry and exit of blood vessels in the glomerulus) and is commonly associated with obesity and hypertension. Also associated with hyperfiltration	Adaptive
**Cellular**	It is the most difficult lesion to identify and is marked by the presence of an increase in cells within the glomeruli and is generally secondary to an inflammatory process	Primary; Adaptive
**Tip-Lesion**	Associated with intense proteinuria, with lesions close to the proximal tubule	Primary

**Table 4:** FSGS remission criteria. Adapted from [22].

Type of Remission	Proteinuria	GFR	Serum Albumin
**Complete Remission (CR)**	<0.3 g/day or <0.3 g/g creatinine	Stable	Disappearance of oedema, normalization of albumin (>3.5 g/dL) and lipids
**Partial Remission (PR)**	0.3–3.5 g/day or 0.3–3.5 g/g creatinine, each with a >50% reduction in albuminuria from baseline	Stable	Disappearance of oedema, normalization of albumin and lipids

**Table 5:** Types of response and possible clinical and laboratory criteria adopted during the treatment of FSGS with GC. Adapted from [32].

Response Types	Clinical and Laboratory Criteria
**Resistance**	Persistence of oedema, hypoalbuminemia, dyslipidemia and 24 h proteinuria >3.5 g
**Recurrence**	After the partial or total response, a new nephrotic outbreak appears (common: two relapses in six months or four relapses in 12 months)
**Corticosteroid dependence**	Two or more relapses during the corticosteroid dose reduction period, or two consecutive relapses, occurring within two weeks of the end of corticosteroid therapy

**Table 6:** Summary of conventional pharmacological treatment for FSGS.

Type of Therapy	Pharmaco-Therapeutic Classification	Examples of Drugs	Drug Action in FSGS	References
**Symptomatic**	Angiotensin-Converting Enzyme Inhibitor (ACEi)	Ramipril	Treat blood pressure and reduce proteinuria	[8,53]
	Angiotensin II Receptor Antagonist (ARA)	Telmisartan Ibersartan Sparsentan		[8,54]
	Thiazide Diuretics	Hydrochloro-thiazide; Chlorthalidone	Control oedema and promote fluid excretion, boosting the effects of RAAS inhibitors	[8]
**Immunosuppressive**	Glucocorticoids (GCs)	Prednisolone Methylprednisolone	They reduce inflammation and the immune response to prevent the formation of new scars	[22]
	Calcineurin Inhibitors (ICNs)	Cyclosporine A Tacrolimus	Calcineurin is part of the T cell signaling pathway and participates in the activation of IL-2 production, promoting an immune response in various cell types	[55]
	Antiproliferation and antimetabolic medicines	Mycophenolate mofetil	It inhibits purine synthesis, which reduces the proliferation of T and B cells and, consequently, the immune response and proteinuria	[22,32]
	Biological medicines/Monoclonal antibodies	Baliximab Rituximab	Allow for the elimination of B cells and the interruption of interactions between B cells and T cells that cause proteinuria	[22,44]
	Alkylating agents	Cyclophosphamide	Interferes with DNA replication, causing cellular damage that can reduce the inflammatory and immune response, decreasing the progression of kidney disease and proteinuria	[22]

**Table 7:** Main characteristics of CDs—α, β and γ. Adapted from [28,64].

Cyclodextrin	Alpha (α)	Beta (β)	Gamma (γ)
**Height (nm)**	0.78	0.78	0.78
**Molecular Formula**	C_(36)H(60)O30_	C_(42)H(70)O35_	C_(48)H(80)O40_
**Physicochemical characterization**	Hydrophilic; Homogeneous and crystalline substances	Hydrophilic; Homogeneous and crystalline substances	Hydrophilic; Homogeneous and crystalline substances
**Chemical composition**	6 units of cyclic oligosaccharides	7 units of cyclic oligosaccharides	8 units of cyclic oligosaccharides
**Inner Diameter (nm)**	0.47–0.53	0.60–0.65	0.75–0.83
**Outer Diameter (nm)**	1.46	1.54	1.75

**Table 8:** Effect of CDs—α, β and γ—on the aqueous solubility of cyclosporine A. Adapted from [73].

Cyclodextrin	CE	Solubility (mg/mL) in the Presence of 5% (*w*/*v*) CDs	Solubility (mg/mL) in the Presence of 15% (*w*/*v*) CDs
**α**	0.54	0.76 ± 0.02	4.22 ± 0.59
**β**	0.030	Not Soluble	Not Soluble
**γ**	0.0049	0.062 ± 0.001	0.11 ± 0.00

**Abbreviations: CD** (cyclodextrin); **CE** (complexation efficiency); ***w*/*v*** (weight per volume).

**Table 9:** Drug content and aqueous solubility of tacrolimus and tacrolimus encapsulated in inclusion systems with β-CD. Adapted from [81].

Drug/Inclusion Complex	Drug Content (%*w*/*w*)	Aqueous Solubility (μg/mL)
**Tacrolimus**	N.A	3.05 ± 0.1210
**Tacrolimus with β-CD**	96.84 ± 1.76	14.82 ± 0.8890

**Abbreviations: %*w*/*w*** (weight by weight percentage); **μg/mL** (microgram per milliliter).

**Table 10:** Summary table of studies with CDs and drugs used in FSGS.

Cyclodextrin	Drug/Active Ingredient	In Vitro Study	In Vivo Study	Results	References
**α**	Cyclosporine A	“Cyclodextrin complexes of a globular protein and a lipophilic oligopeptide: the effect of structure and physicochemical properties”	N.A	The solubility of the drug was increased; It was also found that the drug stabilized	[73]
	Prednisolone	“Prediction of the free energy of binding for cyclodextrin-steroid complexes: phase solubility and molecular dynamics studies”	N.A	The solubility profile of the drug increased	[77]
**β**	Hydrochlorothiazide	“Effect of β-cyclodextrin and different surfactants on solubility, stability, and permeability of hydrochlorothiazide”	N.A	β-Cyclodextrin increased the solubility, permeability and stability of the drug	[75]
		“Combined Approach of Cyclodextrin Complexation and Nanostructured Lipid Carriers For the Development of Pediatric Liquid Oral Dosage Form of Hydrochlorothiazide”	N.A	Hydrochlorothiazide, when encapsulated in the (2-hydroxy)propyl-β-cyclodextrin is released in a more controlled and complete way, compared to when this drug is administered on its own without the use of any type of cyclodextrin complexation system	[80]
	Mycophenolate Mofetil	“Solid Lipid Nanoparticles of Mycophenolate Mofetil: An Attempt to Control the Release of an Immunosuppressant”	N.A	β-Cyclodextrin increased the solubility of the drug and it is more easily released when combined with the β-cyclodextrin	[79]
	Spironolactone	N.A	“In Vivo Investigation of (2-Hydroxypropyl)-β-cyclodextrin Based Formulation of Spirinolactone in Aqueous Solution for Pediatric Use”	The (2-hydroxy)propyl-β-cyclodextrin increased the bioavailability, solubility and dissolution rate of spironolactone. There were no beneficial effects of this cyclodextrin in masking the flavor of oral formulations	[76]
	Cyclosporine A	“Cyclodextrin complexes of a globular protein and a lipophilic oligopeptide: the effect of structure and physicochemical properties”	N.A	The solubility of the drug was increased; It was also found that the drug stabilized	[73]
	Telmisartan	“Long Term Stability and In-vitro release study of telmisartan complex included by hydroxypropyl-beta-cyclodextrin in directly compressed tablet using ion-pair reversed phase high-performance liquid chromatography”	N.A	The dissolution rate of the drug increased; It was also found that due to CD the long-term stability of the drug improved, with no changes in its physical and chemical properties	[78]
	Tacrolimus	“Development and characterization of tacrolimus tablet formulations for sublingual administration”	N.A	There was an increase in the solubility of the drug as well as in the speed of disintegration; Dissolution profiles also showed improvements	[81]
**γ**	Hydrochlorothiazide, Telmisartan and Ibersartan	“Evaluation of γ-cyclodextrin effect on permeation of lipophilic drugs: application of cellophane/fused octanol membrane”	N.A	γ-Cyclodextrin increased the permeability of all the drugs tested. However, this increase in permeability depended on the properties of each drug	[83]
	Hydrochlorothiazide	N.A	“Inclusion complex of hydrochlorothiazide-γ-Cyclodextrin: The effect on aqueous solubility, dissolution rate, bioavailability and the effect on intestinal permeability using Ussing Chamber Technique”	γ-Cyclodextrin increased the aqueous solubility as well as the dissolution rate, bioavailability and permeability of the drug	[84]
	Cyclosporine A	“Cyclodextrin complexes of a globular protein and a lipophilic oligopeptide: the effect of structure and physicochemical properties”	N.A	The solubility of the drug was increased; It was also found that the drug stabilized	[73]
	Prednisolone	“Development of prednisolone-containing eye drop formulations by cyclodextrin complexation and antimicrobial, mucoadhesive biopolymer”	N.A	The solubility and the bioavailability of the drug were increased. In addition, there was an increase in the diffusion of this drug in the corneal cell membrane	[82]
	Methylprednisolone	“Comparison of the complexation between methylprednisolone and different cyclodextrins in solution by 1H-NMR and molecular modelling studies”	N.A	The aqueous solubility of the drug increased	[85]

**N.A**: Not applicable.

## Data Availability

No new data were created or analyzed in this study. Data sharing is not applicable to this article.
